# ARRONAX Cyclotron: Setting up of In-House Hospital Radiopharmacy

**DOI:** 10.1155/2020/1572841

**Published:** 2020-03-10

**Authors:** Aurelien Vidal, Cécile Bourdeau, Mathieu Frindel, Tracy Garcia, Ferid Haddad, Alain Faivre-Chauvet, Mickaël Bourgeois

**Affiliations:** ^1^ARRONAX Cyclotron, 1 Rue Aronnax, 44817 Saint Herblain, France; ^2^ICO Nantes-Nuclear Medicine Department, Boulevard Jacques Monod, 44805 Saint Herblain, France; ^3^Nantes University Hospital-Nuclear Medicine Department, 9 Quai Moncousu, 44093 Nantes, France; ^4^CRCINA, INSERM, CNRS, Université d'Angers, Université de Nantes, Nantes, France

## Abstract

Whilst radiopharmaceuticals have an important role to play in both imaging and treatment of patients, most notably cancer patients, nuclear medicine and radiopharmacy are currently facing challenges to create innovative new drugs. Traditional radiopharmaceutical manufacture can be considered as either a routine hospital production or a large-scale industrial production. The gap between these two practices has meant that there is an inability to supply innovative radiopharmaceuticals for use at the local level for mono- or multicentric clinical trials with satisfactory quality and safety specifications. This article highlights the regulatory requirements in aseptic pharmaceutical processing and in nuclear medicine to be able to locally produce radiopharmaceuticals. We validate the proof-of-concept for an “in-house” hospital-based radiopharmacy including an on-site cyclotron, that can fulfill the conflicting requirements between radiation safety and aseptic processing. The ARRONAX in-house radiopharmacy is currently able to provide sterile and pyrogenic-free injectable radiopharmaceutical compounds for both industrial and institutional clinical trials.

## 1. Introduction

Nuclear medicine is a medical specialty that uses radioactive atoms for diagnosis and/or therapy. Radioactive atoms can be coupled to organic or biologic molecules to create a radiopharmaceutical that has a specific biological target, and therefore allows targeting and delivery of the radioactivity to pathologic tissues. For many years, the variety of available radioisotopes for radiopharmaceutical use was quite limited; however, recent progress in radioisotope production and in the development of new vector molecules has changed the field of nuclear medicine considerably. This is particularly relevant to the management of oncology patients, with a large number and variety of new radiopharmaceuticals reaching the clinical trial stage.

To support this increased need, particle accelerators such as cyclotrons were built around the world to produce various radioisotopes. Due to the physical properties of radioisotopes and the transportation regulation constraints, a network of cyclotrons has been created such that the production site is relatively close to the hospital unit where the radiopharmaceutical is injected into the patients.

As with all injectable medicinal products, radiopharmaceuticals need to be safe, reliable, and consistent, as well as comply with current regulations and guidelines.

As a consequence of the relatively short half-life of some radionuclides and the requirement for aseptic production techniques, small-batch radiopharmaceutical production for clinical trials can be a challenge for healthcare establishments.

The aim of this article is to describe the setting up of an “in-house” production area in a French public hospital (Nantes University Hospital) to support the production of new and innovative radiopharmaceuticals for nuclear medicine clinical trials.

## 2. Materials and Methods

### 2.1. Regulatory Compliance for Radiopharmaceutical Production

Sterile radiopharmaceutical production must comply with both “conventional” sterile pharmaceutical production and nuclear regulatory requirements [1]. With respect to pharmaceuticals, French regulation is directly under the control of international legislation adopted by the European Medicines Agency (EMA) including the International Council for Harmonisation of Technical Requirements for Pharmaceuticals for Human Use (“ICHQ7 Good Manufacturing Practice for Active Pharmaceutical ingredients”) and the World Health Organization Annex 6 (“WHO Good Manufacturing Practices for Sterile Pharmaceutical Products”). These international guidelines were combined by the European Commission and aggregated under the EudraLex guidelines with a specific section on Good Manufacturing Practices (GMP) required for the manufacture of medical radiopharmaceuticals for human use. In addition to the European legal requirements, France also requires adherence to the European Pharmacopoeia (Ph. Eur.) and to all specific monographs encountered during radiopharmaceutical activities, e.g., general monograph on radiopharmaceutical preparations (0125) or the monograph on biological tests (20601).

Locally, our manufacturing approach is also guided by additional reference documents. These relate to the specific state-of-the-art radiopharmacy and hospital requirements (e.g., small batches, early phase clinical trials, and variability in complexity for radiopharmaceutical preparation). As an example, the ARRONAX in-house radiopharmacy of the Nantes University Hospital also bases its practices on various recommendations such as the “PIC/S Guide to Good Practices for the Preparation of Medicinal Products in Healthcare Establishments” of the Pharmaceutical Inspection Cooperation Scheme (PIC/S) or the “Guidelines on Current Good Radiopharmacy Practices (cGRRP) in the Preparation of Radiopharmaceuticals” established by the radiopharmacy committee of the knowledge society European Association of Nuclear Medicine (EANM) [2].

In terms of the radioactive aspects, France follows the “International Atomic Energy Agency (IAEA)” recommendations, transcribed in European regulations under the Euratom directives and applied under French law. In France, transport and delivery of radioactive materials are authorized under the jurisdiction of an independent administrative authority, the “Nuclear Safety Authority (ASN).”

### 2.2. ARRONAX In-House Radiopharmacy Description

The ARRONAX cyclotron (Accelerator for Research in Radiochemistry and Oncology at Nantes Atlantic) is located in Nantes and is a French public asset. It is regulated by a number of stakeholders including the French State (Ministry of Higher Education and Research), the Pays de la Loire regional council, Nantes University, the National Institute for Health and Medical Research (INSERM), the National Center for Science Research (CNRS), the engineering school “Ecole des Mines de Nantes,” the Western Cancer Institute (ICO), and the Nantes University Hospital [3, 4].

**Figure 1:**
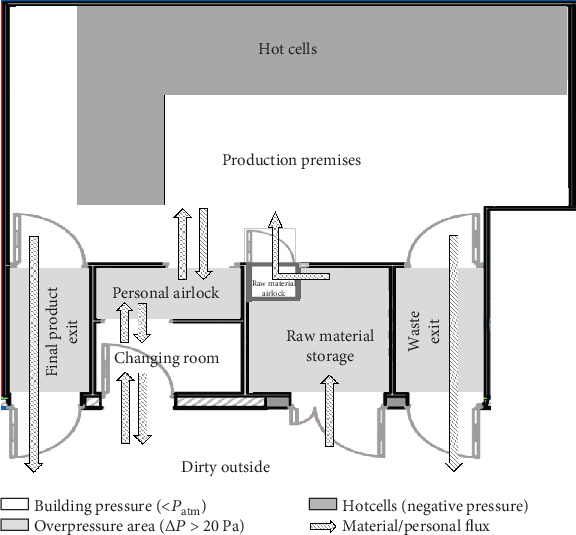
General plan of ARRONAX “in-house” radiopharmacy.

The ARRONAX in-house radiopharmacy is governed by the Nantes University Hospital. The radiopharmacy production premises house and operate both radionucleotide production and pharmaceutical manufacturing aspects in respect of radiation safety regulation and aseptic processing requirements. Access to this room is restricted to qualified and authorized persons. The exterior of the radiopharmacy is considered to be dirty, and the production principle is for a minimal number of personnel to only move from dirty to clean areas following an appropriate change of apparel. In addition, sanitation of radiopharmaceutical production materials at each interface is required. One of the main problems in the design of a radiopharmacy is dealing with the conflicting airflow requirements. Radioprotection requires negative pressure (confinement of gaseous or aerosol discharge), whilst aseptic production requires positive pressure to prevent contamination by microbes. To manage this and satisfy both requirements, we have implemented an overpressure bridge between the dirty outside and the clean area. The production premises is a classified grade D cleanroom in order to minimize microbe entrance and to prevent cross-contamination. Movement of personnel, raw materials, final products, and waste through the radiopharmacy are strictly directional, and are only possible through separate and interlocked entrance/exits.  Figure 1 shows the general plan of the ARRONAX in-house radiopharmacy, the “hot cell” production area, the air pressure within each room, and the direction of material/personal/waste movement.

**Figure 2:**
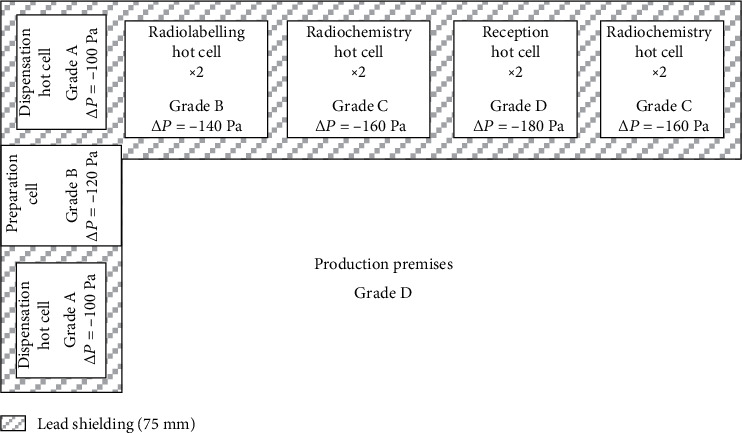
Hot cells of ARRONAX “in-house” radiopharmacy.

For radiosafety reasons, the radiopharmaceutical production steps are conducted in 8 lead shielded isolators called “hot cells.” To prevent the risk of radioactive contamination of the staff with gaseous or aerosol discharge, these hot cells are placed under negative pressure. The ARRONAX in-house radiopharmacy hot cells were manufactured and installed by Von Gahlen (Zevenaar, Netherlands) and are designed for the various operations required in a cyclotron radiopharmacy (Figure 2).

Overall, the hot cell facility is subdivided into two categories, with 3 hot cells for radiochemistry and 5 hot cells for production of the radiopharmaceutical.

The most “dirty” hot cell is classified as a grade D cleanroom with a negative pressure of -180 Pa compared to the production premises. This hot cell is used for the reception of the irradiated materials and is connected to the cyclotron irradiation vault by a pneumatic system enabling transfer of the solid target by a shuttle system. On both sides of this reception hot cell, there are 2 radiochemistry hot cells classified as grade C cleanrooms, with a negative pressure of -160 Pa compared to the production premises. These are dedicated for the extraction of radionuclides from the solid target, their purification, and their conditioning for future radiolabelling in the radiopharmaceutical process. Work in the grade D and C hot cells is conducted with telemanipulators, and solid materials are transferred through airlocks.

For the radiopharmaceutical production, the ARRONAX in-house radiopharmacy has 2 radiolabelling hot cells placed one on top of the other, where the radionuclide (in liquid solution) is radiolabelled to an organic or biologic molecule to obtain the radiopharmaceutical compound. These 2 hot cells are classified as grade B cleanrooms with a negative pressure of -140 Pa compared to the production premises. The different steps of this process are performed by automated systems controlled by a dedicated computer system. After these radiolabelling steps, the radiopharmaceutical compound can be transferred by a capillary system to 2 dispensing hot cells. Here, the compound is measured by a calibrated dose calibrator (ionization chamber), formulated, and aseptically dispensed in the final vial. Because of the aseptic requirements, these 2 hot cells are classified as grade A under vertical laminar airflow with a negative pressure of -100 Pa compared to the production premises [5]. Between these 2 hot cells is a preparation cell to allow aseptic entry of sterile materials required for the final dispensation steps. This preparation cell is not shielded (no radioactivity inside) and is classified as grade B with a negative pressure of -120 Pa compared to the production premises. Appropriate installation qualification (IQ) and operational qualification (OQ) demonstrated the suitability of all the hot cells to be suitable for their assigned tasks. The performance qualification (PQ) of the hot cells was demonstrated during the feasibility study and the process validation for each radiopharmaceutical produced in the radiopharmacy [6].

The 2 radiolabelling hot cells are equipped with specialized and dedicated automated synthesis equipment. Currently, the ARRONAX “in-house” radiopharmacy has two radiosynthesis devices provided by Eckert & Ziegler (Berlin, Germany). The upper radiolabelling hot cell is equipped with a Modular-Lab Standard® automated system, and the lower one is equipped with a Modular-Lab PharmTracer® system.

The Modular-Lab Standard® system is a versatile and flexible tubing-based system that is suitable for small-scale early clinical trials and customized radiosyntheses. The Modular-Lab PharmTracer® is a cassette-based automation system particularly suited to later stage clinical trials and routine production as it has a closed transfer system linked to a sterile and single-use cassette. Both systems are driven by Modular-Lab Software®, which uses a GMP-compliant graphic user interface and complies with good automated manufacturing practice (GAMP) and to FDA 21 CFR part 11 regulations. Both radiosynthesis devices conformed to IQ and OQ requirements after their installation in the hot cells, and this included various technical parameters (function of solenoid valves, heating of the reactor modules, pressure control for pharmaceutic gases, and control of syringe volume and flow rate). The automated radiosynthesizers are subject to a yearly preventive maintenance contract during which they are recalibrated. Radiosynthesizer PQ was demonstrated during the feasibility study and the process validation for each radiopharmaceutical produced in the radiopharmacy.

One of the grade A dispensing hot cells is equipped with an Open Vial Dose Dispenser (OVDD) purchased from Von Gahlen (Zevenaar, Netherlands). This vial dispenser is an automated system which performs the formulating, fractioning, and aseptic filling of the final bulk product which comes from the radiosynthesizer via capillary tubing. OVDD is a single-use cassette-based automated system which performs final sterilizing filtration, complete vial filling, and automatic septum/cap crimping. OVDD has the same quality aspects (i.e., maintenance contract, IQ, OQ, and PQ) as the two radiosynthesizers.

The ARRONAX in-house radiopharmacy also performs as a quality control laboratory (QC lab). In accordance with GMP requirements, the staff responsible for QC are independent of the production staff. The QC lab is equipped with an HPLC system (Shimadzu; Kyoto, Japan), an Agilent GC system (Santa Clara, USA), a Perkin Elmer thin-layer radiochromatograph (Wellesley, USA), and an Endosafe® nexgen-PTS® acquired from Charles River Laboratories (Wilmington, USA) to assay for endotoxin levels in the final product. All analytical devices were subject to IQ and OQ at the time of installation and are monitored under an annual preventive maintenance contract. The PQ of these instruments is performed during the validation of analytical procedures for each measured parameter of a radiopharmaceutical compound.

## 3. Results

Before starting a radiopharmaceutical preparation, the outside surfaces of all raw materials and consumables are properly decontaminated with quaternary ammonium solution or isopropyl alcohol. All vials containing radioactivity are placed in lead-shielded containers. Prior to starting work, radiopharmacy staff wash their hands thoroughly; remove their clothes; and change into clean low-lint overalls, overshoes, hair cover, mask, and gloves. Just before entrance, gloves are rubbed with hydroalcoholic solution. Adherence to the various standard operating procedures (e.g., staff entrance process, direction of raw material and consumable movement, frequency of room biocleaning, interlocking of the airlocks, and setting up of the overpressure barrier) results in good conformity to the cleanroom grade D classification requirements of the radiopharmacy premises. For the *at rest* air particle monitoring, the number of sampling points are determined by the ISO 14644 norm [7] and are, respectively, 9 for the production premises, 2 for the final product exit, 2 for the waste exit, 1 for the personal airlock, 2 for the changing room, and 3 for the raw material storage. For bacteriological monitoring, we monitor ground contamination using 55 mm diameter contact plates and air sample monitoring that follows the ISO 14698 norm [8]. For the radiopharmacy premises, temperature, differential pressure, hygrometry, particle counting (0.5 and 5.0 *μ*m), and radioactive levels are monitored in real time and are controlled by a building monitoring system. The mean results (±SD) of the environmental and bacteriological particle monitoring for the 2 last years are presented in Table 1.

Concerning the hot cells, differential pressure, temperature, hygrometry, radioactive level, and air velocity in the exhaust are monitored for all the hot cells in real time. For both grade A dispensing hot cells, continuous monitoring of air velocity under the vertical laminar airflow is performed. Bacteriological and environmental particle monitoring is conducted at rest for grade B to D hot cells and at rest and in operation for both grade A distribution hot cells. The mean results (±SD) of the bacteriological and environmental particle monitoring for the 2 last years are presented in Table 2.

For clinical trials, each new radiopharmaceutical production is validated by a feasibility and repeatability radiolabelling process (3 runs at least). These tests are used to fulfill the PQ for the automated radiochemical production. To assess the aseptic process PQ, at least 3 runs are performed whereby the final filtration step is disconnected. This allows the extemporaneous bacteriological and endotoxin levels in the radionuclides to be analysed, and it is performed by an external subcontractor laboratory. The other steps of the process are carried out in accordance to the master batch record. The global aseptic process (automated and manual) is validated by a “media-fill approach” where the raw materials are replaced by bacteriological culture media during critical steps. To date, all radiopharmaceuticals for clinical trials produced within the ARRONAX in-house radiopharmacy have been validated using these complementary approaches and have demonstrated the capacity to prepare sterile radiopharmaceutical compounds which comply to all the physiochemical specifications in accordance with the IMPD requirements. Both automated radiosynthesizers (Modular-Lab PharmTracer® and Modular-Lab Standard®) allowed for closed vial filling and final sterilization. After each synthesis, the filter integrity is tested by an overpressure monitor directly managed by the system. Concerning the OVDD automation, which performs open vial filling, the filter integrity is tested by an automatic bubble point test after each batch.

The final radioactive dose of a radiopharmaceutical compound is given in Becquerel (or in the former Curie units for certain countries). Classically, this radioactive dose is measured by a gaseous ionization detector called a “dose calibrator.” Due to physical and metrological reasons, a dose calibrator needs to be independently calibrated for each radionuclide and for each geometry (vial, syringe, etc.). For routine nuclear medicine and conventional radionuclides, a great variety of radioactive calibration sources exist, and it is quite easy to obtain a calibration certificate for a given radionuclide. For innovative radionuclides produced in a research cyclotron such as the ARRONAX, no calibration source is available, and therefore, the dose calibrator needs to be calibrated “in-house.” To perform this, the ARRONAX in-house radiopharmacy performs an intercalibration measurement with a HPGe semiconductor gamma spectrometer calibrated with multigamma emitters provided by a COFRAC-certified metrologic laboratory.

Concerning the QC lab, all equipments are certified and the analytical methods follow respective European Pharmacopoeia monographs concerning chromatographic separation techniques (PhEur 2.2.46), thin-layer chromatography (PhEur 2.2.27), gas chromatography (PhEur 2.2.28), liquid chromatography (PhEur 2.2.29), and size exclusion chromatography (PhEur 2.2.30). For the analytics apparatus (HPLC, GC, and TLC radiochromatograph), the PQ is performed during the validation of analytical procedures in conformity with ICH Q2 recommendations concerning the demonstration of suitability for the intended purpose. Typical validation characteristics consider various items such as specificity, linearity, range, accuracy, precision, detection, and quantitation limits. Final product extemporaneous endotoxin dosage is measured by the QC lab in accordance with the chromogenic kinetic method described in the bacterial endotoxin monograph (PhEur 2.6.14), and for each new radiopharmaceutical production within the ARRONAX in-house radiopharmacy, the maximum validated dilution is determined.

## 4. Discussion and Conclusion

The design, commission, and operation of the ARRONAX in-house radiopharmacy comply with the bacteriological and environmental particulate requirements required for the production of sterile radiopharmaceuticals for use in early phase clinical trials in a hospital nuclear medicine department. Environmental monitoring 2 years after the operation began revealed values under the regulatory standard for each of the rooms and hot cells. We observed only one elevated measurement in one hot cell, and it reached several alert thresholds (internally set at 2/3 of the regulatory thresholds) after mechanical maintenance of the hot cells. These were restored to normal after a standard biocleaning of the room and utilities. As a result of this experience, we schedule a systematic large-scale biocleaning following any maintenance work in order to prevent any aseptic failure.

On the radioactive side of the production facility, waste management and fabrication line cleaning are managed around the radioactive half-life of the radiopharmaceuticals and the production schedule requirements. For the workers in the radiopharmacy unit, the “As Low As Reasonably Achievable” (ALARA) radiosafety philosophy is followed, and the total annual exposure doses conform to the French B category of radioactive workers (less than 6 mSv to the body, 15 mSv to the eyes, and 150 mSv to the hands).

The ARRONAX in-house radiopharmacy is authorized by the local Regional Health Agency (ARS Pays de la Loire) for the preparation of radiopharmaceutical compounds dedicated to clinical trials in nuclear medicine, and by the French Nuclear Safety Authority (ASN) for the operation of a particle accelerator and for the production and distribution of radiopharmaceuticals dedicated to clinical trials in nuclear medicine. These authorizations cover the facility and utilities. For each new radiopharmaceutical involved in a clinical trial, the process validation and the equipment qualification are checked and validated by the French National Agency for Medicines and Health Product Safety (ANSM) during the Investigational Medicinal Product Dossier (IMPD) instruction. To date, the ARRONAX in-house radiopharmacy has produced radiopharmaceuticals for clinical trials conducted by both industrial and institutional clients and both monocentric and multicentric investigations (Table 3).

The increment of quality assurance requirement for staff, premises, and equipments with full qualification and process validation leads hospital radiopharmacy to meet pharmaceutic industrial requirements for a clinical trial. Nuclear medicine is currently undergoing significant advances due to the development of new diagnostics and therapeutics. The ARRONAX in-house radiopharmacy is a validated proof-of-concept facility capable of producing quality innovative radiopharmaceutical compounds on-site.

## Figures and Tables

**Table 1:** Environmental monitoring of the radiopharmacy premises.

	Ground sample (CFU/plate)		Air sample (CFU/m^3^)		0.5 *μ*m particles/m^3^		5.0 *μ*m particles/m^3^	
	Mean	±SD	Mean	±SD	Mean	±SD	Mean	±SD
Radiopharmacy premises	3.2	2.8	9.5	9.0	37,865	1,798	1,493	420
Raw material storage	7.1	7.0	29.4	18.2	147,292	8,789	12,172	1,778
Changing room	17.4	3.8	15.8	12.3	150,930	9,441	12,564	1,504
Personal airlock	5.3	5.2	23.0	15.2	75,509	3,690	4,078	985
Final product exit	6.1	3.8	21.5	16.2	93,739	4,609	10,667	1,609
Waste exit	9.9	7.3	24.3	6.1	108,054	5,519	18,146	2,561

**Table 2:** Environmental monitoring of the hot cells.

	Ground sample (CFU/plate)		Air sample (CFU/m^3^)		0.5 *μ*m particles/m^3^		5.0 *μ*m particles/m^3^	
	Mean	±SD	Mean	±SD	Mean	±SD	Mean	±SD
Reception hot cells	7	2	35	15	248,895	32,565	1534	474
Radiochemistry hot cells (×2)	4	1	14	11	105,358	32,537	489	141
Radiolabelling hot cells (×2)	2	1	4	1	2,183	1,196	12	5
Preparation cell	1	0.5	0	0	105	19	2	1
Distribution hot cells (×2)	0	0	0	0	7	1	1	1

**Table 3:** Radiopharmaceuticals for clinical trials currently produced by ARRONAX hospital radiopharmacy.

Radiopharmaceuticals	Promotion	Study start date	Clinicaltrials.gov identifier
^177^Lu-3BP227	Industrial	May 2018	NCT03525392
^177^Lu-OPS201	Industrial	Nov 2018	NCT02592707
^64^Cu-ATSM	Institutional	May 2019	NCT03951337

## Data Availability

The data used to support the findings of this study are included within the article.
